# Triglyceride–Glucose Index Modifies Mortality Risk Across Body Mass Index Strata in Critically Ill Patients: A Retrospective Cohort Analysis of the MIMIC-IV Database

**DOI:** 10.3390/jcm15103685

**Published:** 2026-05-11

**Authors:** Yi Hu, Fan Ping, Wei Bao, Fei Chen, Yian Yao, Hungchen Lin, Zi Ye, Jun Qian, Chengxing Liu, Deqiang Yuan, Kangwei Wang, Yan Lai, Xuebo Liu

**Affiliations:** Department of Cardiology, Tongji Hospital, School of Medicine, Tongji University, Shanghai 200333, China; 2411208@tongji.edu.cn (Y.H.); 1910825@tongji.edu.cn (F.P.); 2411207@tongji.edu.cn (W.B.); riverapt@126.com (F.C.); yaoyian2004@126.com (Y.Y.); 2432569@tongji.edu.cn (H.L.); leafapple8848@hotmail.com (Z.Y.); jgsqianjun@tongji.edu.cn (J.Q.); 2111623@tongji.edu.cn (C.L.); 2031155@tongji.edu.cn (D.Y.); 2311035@tongji.edu.cn (K.W.)

**Keywords:** critically ill, insulin resistance, triglyceride-glucose index, weight management

## Abstract

**Background**: In critically ill patients, the non-linear relationship between body mass index (BMI) and survival has persisted across studies, but whether it is affected by the triglyceride–glucose (TyG) index is unclear. **Methods**: We extracted critically ill patients without diabetes mellitus from the Medical Information Mart for Intensive Care (MIMIC)-IV database and categorized them into T1, T2, and T3 groups according to the TyG index tertiles, with the primary outcome of 365-day all-cause mortality. We used Kaplan–Meier (KM) survival curves, COX regression analyses, and restricted cubic spline curves (RCS) to assess the effect of TyG on the risk of BMI-related mortality. Estimation of optimal change points using segmented linear models. **Results**: The final analytic cohort comprised 6933 critically ill patients with a median age of 66 (54, 76) years, showing male predominance (60.9%). KM curves showed a lower protective effect of obesity in the high-TyG group. COX regression analyses showed that overweight and obesity were associated with lower 365-day all-cause mortality in the T1 and T2 groups and not in the T3 group. RCS analyses showed a U-shaped association between BMI and 365-day all-cause mortality in the T1 and T2 groups and a J-shaped association in the T3 group. The optimal BMI change points were 33 kg/m^2^ (T1), 32.5 kg/m^2^ (T2) and 29.7 kg/m^2^ (T3). **Conclusions**: Our findings demonstrate that the protective effect of obesity was significantly reduced in patients with high TyG levels. Consequently, it is necessary to consider the TyG index in the weight management of critically ill patients.

## 1. Introduction

Obesity has been more common since 1975, and its coexistence with many chronic diseases has become a major health hazard [1,2]. It is estimated that 34.9% of critically ill patients are overweight and 20% are obese [3,4]. Interestingly, an increasing amount of study data and meta-analyses points to a paradoxical relationship in critically ill patients known as the “obesity paradox”: people with overweight or obese seem to die at a lower rate than people of normal weight [5,6,7]. There are also studies that argue that the term “paradox” is inaccurate to describe this ambivalent relationship [8], and that it should be understood that different individuals have different “ideal weights” [9]. In any case, current epidemiologic data are still insufficient to fully elucidate the pathophysiologic mechanisms underlying this paradoxical association. A deeper understanding of this complex association in critically ill patients can inform personalized interventions to improve prognosis through risk stratification.

Insulin resistance (IR), defined as reduced sensitivity to the metabolic effects of insulin, may influence the obesity paradox. Some researchers have attributed the obesity paradox in cardiovascular disease to IR and abnormalities in lipolysis [10]. It has been shown that IR significantly reduces the survival advantage that obesity may confer on post-stroke populations [11]. Furthermore, it has been observed that in patients with chronic heart failure [12] and acute coronary syndromes [13], the phenomenon of the obesity paradox is only evident in non-diabetic patients, which also may be related to IR. The gold standard for assessing insulin sensitivity is the hyperinsulinemic–euglycemic clamp test [14], which is limited by its high cost and complexity, making it difficult to expand clinically. The triglyceride–glucose (TyG) index is a convenient, low-cost surrogate marker of insulin resistance that is increasingly used in epidemiological and risk-stratification research [15]. Although some recent studies combining TyG index with body mass index (BMI) have found that low TyG-BMI levels are associated with increased mortality in critically ill patients associated with some diseases and attributed this to the obesity paradox [16,17,18], it is still unclear whether TyG index affects the obesity paradox in critically ill patients. In other words, it is not clear whether there are different ideal body weights for critically ill patients with different TyG levels. In addition, given that diabetes mellitus (DM) has demonstrated the elimination of the obesity paradox in some studies [12,13,19] and the complex metabolic disorders and medication use in DM, the selection of a non-DM population should provide a clearer representation of the impact of TyG on the obesity paradox.

Therefore, we aimed to investigate whether varying TyG levels modify the BMI–survival relationship in critically ill patients without DM. The findings may inform metabolic health-guided risk stratification and precision weight management strategies for this population.

## 2. Materials and Methods

### 2.1. Data Source

The present study is a retrospective study of critically ill patients in non-critical care medicine with data from a large de-identified database, the Medical Information Mart for Intensive Care (MIMIC)-IV (version 3.1) [20,21]. MIMIC-IV v3.1 was released in October 2024, which currently collects clinical data on patients admitted to Beth Israel Deaconess Medical Center between 2008 and 2022, including more than 65,000 patients admitted to the intensive care unit (ICU) and more than 200,000 patients admitted to the emergency department. The database systematically contains multidimensional clinical information on patients, including demographic characteristics, diagnosis, laboratory tests, procedures, medications, and survival status. After completing the training and signing the data use agreement, we have access to the MIMIC-IV database through PhysioNet [22]. As the database did not contain proprietary information and the patients were anonymous, informed consent was waived.

### 2.2. Study Design and Population

**Figure 1:**
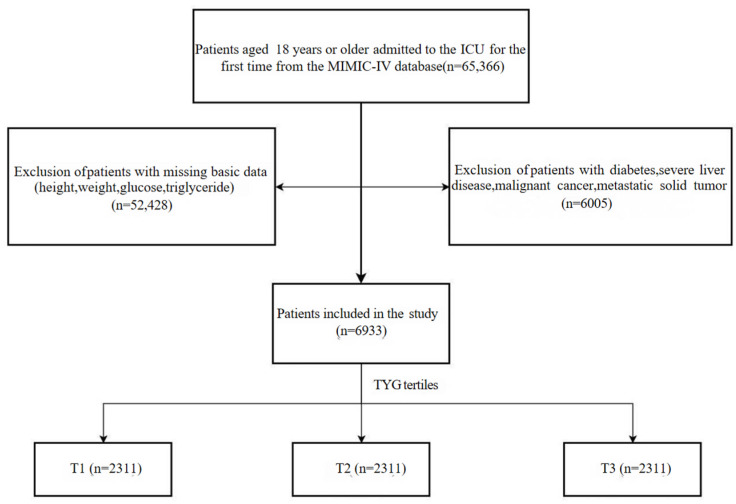
Flowchart of the selection of patients.

All patients admitted for the first time in the ICU were included in the screening. Further exclusion criteria were as follows: (1) patients lacking height, weight, triglycerides (TG), and fasting blood glucose (FBG); (2) patients with DM, severe liver disease, malignant cancer, and metastatic solid tumor. A final total of 6933 patients were included and divided into three groups according to the tertiles of the TyG index (Figure 1).

### 2.3. Data Extraction

We used Navicat Premium (version 16.0.13) to extract data using structured query language (SQL). The extracted data contained (1) demographic data: age, sex, race, height, and weight; (2) burden of disease: cerebrovascular disease, chronic pulmonary disease, congestive heart failure (CHF), hypertension, mild liver disease, myocardial infarction (MI), peripheral vascular disease (PVD), peptic ulcer disease, rheumatic disease, renal disease, and stroke; (3) vital signs: systolic blood pressure (SBP), diastolic blood pressure (DBP), mean blood pressure (MBP), respiratory rate (RR), and heart rate (HR); (4) medication: angiotensin-converting enzyme inhibitors/angiotensin receptor blockers (ACEI/ARB), clopidogrel, ticagrelor, beta blockers, calcium channel blockers (CCB), diuretics, amiodarone, insulin, statins, heparin, and warfarin; (5) laboratory data: SpO_2_, anion gap, calcium, bicarbonate, blood urea nitrogen (BUN), chloride, creatinine, sodium, potassium, glucose, prothrombin time (PT), partial prothrombin time (PTT), TG, red blood cell (RBC), platelet, white blood cell (WBC), glasgow coma scale (GCS), sequential organ failure assessment (SOFA), and simplified acute physiology score (SAPSII); (6) length of stay (LOS): LOS in hospital and LOS in ICU; outcome: 180-day mortality and 365-day mortality. Most blood test results are based on the first measurements obtained within 6 h of the patient’s admission to the ICU and within 24 h of hospital admission. Specifically, TG and FBG levels are based on the first measurements taken between 3:00 a.m. and 8:00 a.m. Multiple imputation was used for variables with less than 20% missing values; variables with more than 20% missing values were excluded.

### 2.4. TyG and BMI

The TyG index was calculated as ln [FBG (mg/dL) × TG (mg/dL)/2]. BMI is calculated by dividing the weight (kg) by the square of the height (m). BMI was classified into the following groups in accordance with the World Health Organization (WHO): underweight group (<18.5 kg/m^2^), normal group (18.5–24.9 kg/m^2^), overweight group (25.0–29.9 kg/m^2^), obese I group (30.0–34.9 kg/m^2^), obese II group (35.0–39.9 kg/m^2^), and obese III group (≥40.0 kg/m^2^). Due to the extremely small sample size in the underweight group (*n* = 192), which resulted in insufficient statistical power after grouping, this group was included in the analysis only when BMI was treated as a continuous variable. Additionally, in sensitivity analyses involving changes in BMI subgroups, the non-overweight/obese group (≤24.9 kg/m^2^) served as the control, and the obese group (≥30.0 kg/m^2^) was included as a separate group in the analysis.

### 2.5. Outcomes

The primary outcome of this study was 365-day all-cause mortality. Only 180-day all-cause mortality was used as an outcome in a sensitivity analysis.

### 2.6. Statistical Analysis

In instances where continuous variables conformed to a normal distribution, they were expressed as the mean ± standard deviation (SD), with differences between groups being calculated using one-way analysis of variance (ANOVA). When continuous variables did not conform to a normal distribution, they were expressed as the median and interquartile range (IQR), with differences between groups being calculated using the Kruskal–Wallis test. Categorical variables were expressed as numbers (proportion), with differences between groups being calculated using the chi-square test.

Kaplan–Meier (KM) survival curves were used to explore the incidence of the primary outcome across BMI groups at different TyG levels. Multivariate Cox regression was used to assess the relationship between each BMI group and 365-day mortality at different TyG levels. The normal group was used as a control. Covariates were carefully selected based on baseline and study context and included age, sex, race, SOFA, SAPSII, PT, BUN, WBC, potassium, hypertension, stroke, renal disease, insulin, statin, and diuretics. To look for a potential non-linear relationship between BMI and 365-day mortality at different TyG levels, we performed restricted cubic spline (RCS) analyses with BMI as a continuous variable. If a non-linear relationship existed, the change point was estimated using a segmented linear model, and a two-piecewise Cox regression was applied to either side of the change point to further investigate the relationship between BMI and 365-day mortality at different TyG levels. This study also analyzed subgroups according to sex, age (≤65 years or >65 years), and race. All statistical tests were two-sided, and *p* < 0.05 was considered statistically significant. All statistical analyses were performed using R software (version 4.4.2, R Foundation for Statistical Computing, Vienna, Austria).

### 2.7. Sensitivity Analysis Plan

To assess the stability of the results, additional analyses were performed using different TyG subgroups, BMI subgroups, or different endpoints, respectively. Different TyG groups were identified based on TyG dichotomization; non-overweight/obese, overweight, and obese groups were used as different BMI subgroups, and 180-day all-cause mortality was selected as a different outcome. In addition, special analyses were conducted for people with cardiovascular diseases, including myocardial infarction, heart failure, stroke, hypertension, and cerebrovascular diseases.

## 3. Results

### 3.1. Baseline Characteristics

The median age of the 6933 patients was 66 (54.76) years, 60.9% were male, and 65.8% were white. Baseline characteristics were analyzed according to the TyG index stratification (T1: <8.62; T2: 8.62–9.20; T3: >9.20) at the time of admission to the ICU. Key characteristics are presented in Table 1. Detailed demographic data, disease burden, vital signs, medication use, laboratory tests, length of hospital stay, and outcome data are provided in Supplementary Table S1. Patients with the highest TyG index were younger, more likely white or male, had lower prevalences of cerebrovascular disease and CHF, higher 180-day versus 365-day all-cause mortality, lower rates of ACEI/ARB, amiodarone, statin and warfarin use, and higher rates of beta blocker, diuretic, insulin and heparin use. They had higher levels of BMI, HR, RR, DBP, anion gap, BUN, creatinine, potassium, glucose, TG, RBC, platelets, WBC, LOS in hospital and ICU, and lower levels of SpO_2_, calcium, bicarbonate, chloride, PT, and PTT.

### 3.2. Association Between BMI and 365-Day All-Cause Mortality Stratified by TyG

K-M curves showed that in groups T1 and T2, the normal group had the lowest survival rate compared to the groups with abnormal BMI (overweight, obese I, and obese II–III), and in group T3, the normal group had the lowest survival rate compared to the overweight and obese I group. No differences in survival were observed between any of the other groups (Supplementary Figure S1). As shown in Table 2, we conducted further Cox regression analyses adjusting for covariates using the normal BMI group as a reference. In group T1, overweight, obesity class I and obesity classes II–III were associated with a significant reduction in 365-day all-cause mortality compared with patients with normal BMI, with adjusted hazard ratios (HRs) and 95% confidence intervals (CIs) of 0.74 (0.58–0.94), 0.62 (0.45–0.85), and 0.67 (0.46–0.99), respectively; in group T2, the only obesity class I was significantly associated with a reduction in 365-day all-cause mortality, with an adjusted HR and 95% CI of 0.66 (0.49–0.90); and in group T3, none of the abnormal BMI groups were significantly associated with 365-day all-cause mortality.

### 3.3. Detection of Non-Linear Relationships

**Figure 2:**
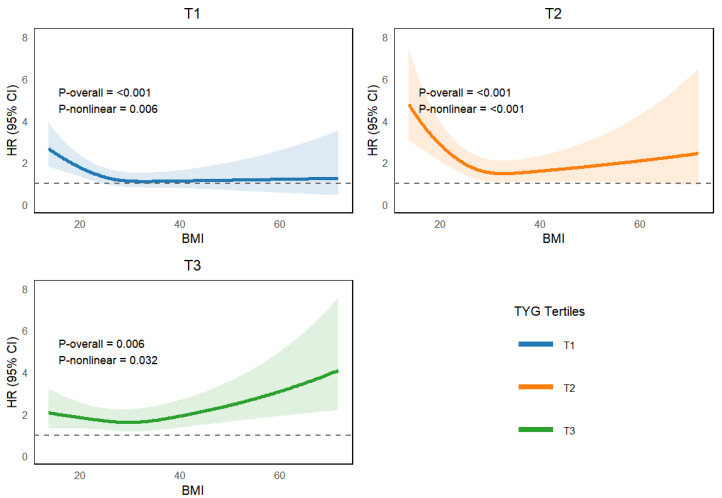
Non-linear relationship between body mass index and 365-day all-cause mortality.

The RCS curves show a non-linear relationship between BMI as a continuous variable and 365-day all-cause mortality in the different TyG subgroups (Figure 2). BMI and 365-day all-cause mortality had a U-shaped association in the T1 and T2 groups and a J-shaped association in the T3 group. Next, the relationship between BMI and 365-day all-cause mortality was further investigated by combining the Cox regression model with a two-piece Cox regression model (both log-likelihood ratios *p* < 0.05) (Table 3). The change points for BMI in the T1, T2, and T3 groups were 33, 32.5, and 29.7, respectively. In either group T1 or T2, BMI was a protective factor for 365-day all-cause mortality when BMI < 33 or BMI < 32.5, with HRs with 95% CIs of 0.956 (0.934–0.977) or 0.942 (0.920–0.966), and there was no significant association between BMI and the risk of death when BMI was greater than or equal to the change point. However, in the T3 group, when BMI ≥ 29.7, BMI was a risk factor for 365-day all-cause mortality (HR 1.023; 95% CI 1.009–1.037), whereas when BMI was <29.7, no significant protective effect of BMI was observed.

### 3.4. Subgroup Analysis

**Figure 3:**
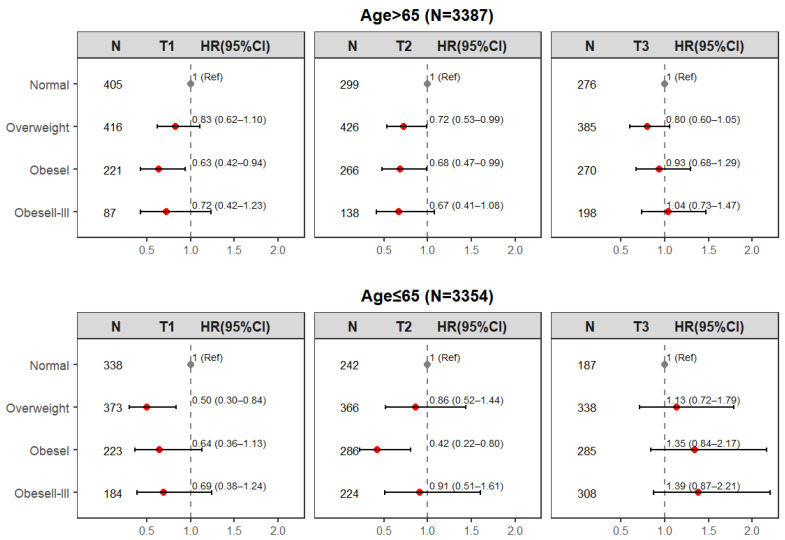
Relationship between body mass index and 365-day all-cause mortality stratified by age. The black circle represents the reference group (Normal BMI; HR = 1.0), and the red circles represent the hazard ratios (HRs) for the comparison groups (Overweight, Obese).

In different subgroups, we explored whether the association between BMI and 365-day all-cause mortality was still influenced by TyG. The results of the subgroup analyses for age and race were similar to the results for the total population (Figure 3, Supplementary Figure S2). In the older, non-older and white, and non-white groups, overweight or obesity had a protective effect on 365-day all-cause mortality in the T1 or T2 groups, but no significant effect in the T3 group. However, some differences emerged in the results of the analyses of the sex subgroups (Supplementary Figure S3). In the male subgroup, overweight protected against 365-day all-cause mortality in three groups, whereas obesity protected only in the T1 and T2 groups; in the female subgroup, the HRs for overweight and obesity were higher in the T3 group than in the T1 and T2 groups, but there was no statistically significant difference in any of the three groups.

### 3.5. Sensitivity Analysis

The results remained unchanged in sensitivity analyses using TyG dichotomous grouping, across BMI groups, and when including 180-day all-cause mortality as an outcome (Supplementary Tables S2–S4). Analyses that included only patients with cardiovascular disease yielded similar results (Supplementary Table S5).

## 4. Discussion

This study investigated whether the relationship between BMI and all-cause mortality was influenced by TyG in 6933 critically ill patients without DM. Our results showed that overweight and obesity were protective in the group with the lowest TyG, obesity class I was protective in T2 groups, and overweight and obesity were not protective in the group with the highest TyG. The lowest value of all-cause mortality in the highest TyG group was 29.7 kg/m^2^, which was significantly lower than the lowest values in the T1 and T2 groups (33 kg/m^2^, 32.5 kg/m^2^). Our study suggests that it is necessary to take TyG levels into account when managing weight in critically ill patients without DM.

People with obesity are not homogeneous groups, such as metabolically healthy obesity (MHO) and metabolically abnormal obesity (MUO), which have attracted much attention in recent years [23]. Previous studies on the obesity paradox in critically ill patients have focused mainly on the association between BMI and mortality, which may ignore the metabolic heterogeneity of people with obesity [24,25]. Although obesity is paradoxically associated with improved survival in critically ill patients, our data suggest that this protective effect is significantly attenuated in patients with elevated TyG, i.e., the effect may be influenced by IR.

The attenuated protective effect of obesity in patients with high TyG levels may involve several potential mechanisms. First, critically ill patients in a high catabolic state may benefit from the nutritional reserves associated with obesity [26]; however, insulin resistance can impair energy utilization through enhanced oxidative stress and mitochondrial dysfunction [27,28,29,30,31,32]. Second, obesity may improve survival in critically ill patients partly through anti-inflammatory adipose tissue remodeling [33,34], but insulin resistance is associated with a shift toward pro-inflammatory macrophage phenotypes and elevated IL-6 levels, potentially counteracting this benefit [35,36,37,38]. Third, adipose tissue secretes beneficial adipokines such as leptin [39,40], and insulin resistance-associated adipose tissue dysfunction may impair the secretion of these protective factors [41,42]. These proposed mechanisms are largely based on preclinical data and remain speculative in the context of critically ill patients.

Interestingly, our subgroup analysis revealed that obesity did not confer protective effects in critically ill women, potentially involving multiple mechanisms. With a median age exceeding 62 years among study participants, the vast majority of women were postmenopausal. First, without the protective effects of estrogen, women’s bioenergetic reserves faced a double blow. On one hand, women lose estrogen’s potent functional barriers—including superior insulin sensitivity, favorable lipid profiles, and anti-inflammatory effects—which instead leads to increased “masculinized” pro-inflammatory visceral fat [43,44]. On the other hand, even at identical BMI values, women possess significantly less lean tissue mass than men [45]. Adequate muscle mass provides essential amino acids during critical moments, offering a buffer against catabolic processes. Research confirms that baseline muscle mass correlates significantly with prognosis in critically ill patients [46]. Furthermore, estrogen is a key hormone regulating adipose tissue metabolism; its deficiency impairs the body’s ability to modulate fat tissue, diminishing obesity’s potential protective effects [47]. Finally, multiple large-scale, long-term epidemiological studies have identified gender differences in “ideal body weight,” with men’s threshold inherently higher than women’s [48,49,50]. Critical care settings may amplify these inherent physiological differences. However, these sex-stratified findings should be interpreted with caution, given the limited statistical power in the female subgroup and the post hoc nature of this analysis. The proposed mechanisms require dedicated prospective investigation.

It should be emphasized that the obesity paradox is not that the fatter you are, the better you are, but that there is an individualized ideal weight. Healthy scientific weight loss may still be beneficial. Several observational studies on bariatric surgery have shown that weight loss reduces hospitalization and mortality in patients with heart failure and also improves cardiac function and symptoms in patients with severe heart failure [51]. On the one hand, this is due to the fact that the population undergoing bariatric surgery has an extremely high BMI (mostly > 35 kg/m^2^), and bariatric surgery reduces weight to a protective range. On the other hand, bariatric surgery has been shown to reduce IR, which further enhances the protective effect of obesity [52]. Healthy exercise interventions are also thought to better reduce visceral fat and improve IR thereby counteracting the deleterious effects of obesity [53]. Therefore, considering the link between IR and the protective effects of obesity is beneficial for understanding and developing strategies for weight management, especially in critically ill patients. Their particular pathology may highlight the protective effects of obesity, and in addition, many critically ill patients are not well enough to actively lose weight through healthy lifestyles or bariatric surgery, making better management in conjunction with IR particularly important.

Our study suggests that after stratification based on the TyG index, the observed protective association of obesity with mortality in critically ill patients appears to vary. The identified BMI inflection points represent statistical change points from observational data and should not be interpreted as prescriptive clinical thresholds. These associations are hypothesis-generating and require prospective validation before clinical application. Of course, weight management in critically ill patients is often challenging. For patients with high TyG scores, nutritional strategies should prioritize enhancing insulin sensitivity and controlling metabolic inflammation rather than merely ensuring caloric intake—aiming to amplify the protective effects of obesity to improve prognosis. It should be noted that the TyG index in this study was derived from measurements taken during ICU hospitalization; although fasting values were selected, their levels may still be significantly influenced by the acute disease stress response. This situation has dual implications for the interpretation of the study’s results: on the one hand, it may suggest that acute metabolic disturbances are a key mediator in attenuating the protective effects of obesity; on the other hand, it may lead to misclassification of groups based on true baseline metabolic status, potentially underestimating the true impact of the TyG index. Future studies that simultaneously record baseline and acute-phase TyG values will be able to more clearly distinguish between these two effects.

However, when interpreting the above findings, the inherent challenge of “reverse causality” in observational studies must be squarely addressed. As demonstrated by the nonlinear analysis in this study and the existing literature, the ICU population with a lower BMI exhibits a higher mortality risk [54]. This likely reflects the higher prevalence of sarcopenia, frailty, and depletion of nutritional reserves among underweight patients, all of which are independently associated with poor outcomes in the ICU. More broadly, a lower BMI may be the result of weight loss caused by chronic wasting diseases (such as cachexia or advanced heart failure), rather than the direct cause of death. While this study partially controlled for this confounding by excluding patients with active cancer, retrospective data still struggle to fully distinguish between obesity as an energy reserve and low body weight as a marker of disease-driven wasting. Future prospective studies that systematically collect data on pre-admission weight changes, frailty indicators, and imaging-based body composition will be better able to disentangle this confounding, thereby more precisely clarifying the true impact of body composition on critical illness outcomes under different metabolic contexts.

The study still had some limitations. First, our data were obtained from the MIMIC-IV database, a single-center retrospective study, and we were unable to exclude potential confounders despite multiple sensitivity analyses. Second, the database lacks measurements of body composition, dietary intake, and physical activity levels. Relying solely on BMI values may obscure actual physical conditions such as sarcopenia, and important confounders including frailty, nutritional status, and inflammatory burden are not captured. BMI is an imprecise proxy for body composition that cannot distinguish lean mass from fat mass or visceral from subcutaneous adiposity. The observed associations may therefore partly reflect underlying health status and body composition rather than adiposity per se. Third, due to the limitations of the exclusion criteria, the study population was restricted to critically ill patients without diabetes; therefore, the results may not be generalizable to other populations. Finally, due to data limitations, this study used only baseline BMI and TYG scores and did not account for their dynamic changes, which may affect the accuracy of the results.

## 5. Conclusions

This study found that different TyG levels affect the obesity paradox in critically ill patients without DM. Obesity was significantly less protective and optimal weight was lower in patients with high TyG levels compared to those with low TyG levels. These findings suggest that metabolic status, as indexed by TyG, may modify the BMI–mortality relationship.

## Figures and Tables

**Table 1:** Baseline characteristics.

Variable	T1	T2	T3	*p*-Value
	<8.62 (*N* = 2311)	(8.62–9.20) (*N* = 2311)	>9.20 (*N* = 2311)	
TyG	8.31 (8.08, 8.48)	8.90 (8.76, 9.04)	9.59 (9.37, 9.97)	<0.001
Age, years	69.00 (56.50, 79.00)	67.00 (56.00, 77.00)	62.00 (49.00, 72.00)	<0.001
Sex, male	1370 (59.3)	1388 (60.1)	1462 (63.3)	0.013
Race, white	1547 (66.9)	1590 (68.8)	1422 (61.5)	<0.001
Weight, Kg	77.20 (65.70, 90.00)	81.20 (67.50, 96.25)	86.10 (72.90, 102.00)	<0.001
Height, cm	170.00 (163.00, 178.00)	170.00 (163.00, 178.00)	170.00 (163.00, 178.00)	0.002
BMI, Kg/m^2^	26.73 (23.40, 30.80)	28.16 (24.37, 32.41)	29.44 (25.64, 34.28)	<0.001
GCS	15.00 (14.00, 15.00)	15.00 (14.00, 15.00)	15.00 (14.00, 15.00)	<0.001
SOFA	4.00 (2.00, 6.00)	4.00 (2.00, 7.00)	5.00 (3.00, 8.00)	<0.001
SAPSII	33.00 (26.00, 42.00)	34.00 (26.00, 42.00)	36.00 (27.00, 46.00)	<0.001
180-day mortality, *n* (%)	346 (15.0)	354 (15.3)	438 (19.0)	<0.001
365-day mortality, *n* (%)	410 (17.7)	401 (17.4)	492 (21.3)	0.001

BMI, body mass index; GCS, Glasgow coma scale; SOFA, sequential organ failure assessment; SAPSII, simplified acute physiology score; TyG, triglyceride–glucose.

**Table 2:** Multifactorial Cox regression analysis of 365-day all-cause mortality.

	BMI Group	Event/N	HR (95%CI)	*p*-Value
T1	Normal	176/741	Reference	-
	Overweight	117/804	0.74 (0.58–0.94)	0.014
	Obese I	55/444	0.62 (0.45–0.85)	0.003
	Obese II–III	32/258	0.67(0.46–0.99)	0.049
T2	Normal	130/573	Reference	-
	Overweight	126/765	0.78 (0.61–1.01)	0.058
	Obese I	69/539	0.66 (0.49–0.90)	0.007
	Obese II–III	52/370	0.76 (0.55–1.07)	0.118
T3	Normal	116/433	Reference	-
	Overweight	146/735	0.82 (0.64–1.05)	0.110
	Obese I	107/568	0.92 (0.70–1.21)	0.560
	Obese II–III	113/511	1.04 (0.79–1.38)	0.771

Adjusted for age, sex, race, SOFA, SAPSII, PT, BUN, WBC, potassium, hypertension, stroke, renal disease, insulin, statin, and diuretics.

**Table 3:** Analysis of the threshold effect of BMI on 365-day all-cause mortality.

		HR (95%CI)	*p*-Value	*p* for Log-Likelihood Ratio
T1	Standard linear model	0.973 (0.957–0.990)	0.002	
	Two-piecewise linear model			0.018
	Change point, kg/m^2^	33		
	BMI < 33	0.956 (0.934–0.977)	<0.001	
	BMI ≥ 33	1.012 (0.982–1.043)	0.439	
T2	Standard linear model	0.974 (0.960–0.991)	0.002	
	Two-piecewise linear model			<0.001
	Change point, kg/m^2^	32.5		
	BMI < 32.5	0.942 (0.920–0.966)	<0.001	
	BMI ≥ 32.5	1.021 (0.993–1.049)	0.143	
T3	Standard linear model	1.011 (0.999–1.023)	0.062	
	Two-piecewise linear model			<0.001
	Change point, kg/m^2^	29.7		
	BMI < 29.7	0.981 (0.952–1.010)	0.200	
	BMI ≥ 29.7	1.023 (1.009–1.037)	0.001	

Adjusted for age, sex, race, SOFA, SAPSII, PT, BUN, WBC, potassium, hypertension, stroke, renal disease, insulin, statin, and diuretics.

## Data Availability

Restrictions apply to the availability of these data. Data were obtained from the Medical Information Mart for Intensive Care (MIMIC)-IV database (version 3.1) on PhysioNet. Researchers can apply for credentialed access through the official website. Requests to access these datasets should be directed to https://physionet.org/content/mimiciv/3.1/ (accessed on 30 October 2025).

## Supplementary Materials

The following supporting information can be downloaded at https://www.mdpi.com/article/10.3390/jcm15103685/s1. Figure S1: Kaplan-Meier survival analysis curves for 365-day all-cause mortality; Figure S2: Relationship between body mass index and 365-day all-cause mortality stratified by race; Figure S3: Relationship between body mass index and 365-day all-cause mortality stratified by sex; Table S1: Baseline characteristics; Table S2: COX regression analysis of 365-day all-cause mortality stratified by TyG dichotomy; Table S3: COX regression analysis of 365-day all-cause mortality in the combined obesity group; Table S4: COX regression analysis of 180-day all-cause mortality rate; Table S5: COX regression analysis of 365-day all-cause mortality in patients with cardiovascular diseases.

## Abbreviations

The following abbreviations are used in this manuscript:

IR	insulin resistance
TyG	triglyceride–glucose
BMI	body mass index
DM	diabetes mellitus
MIMIC	Medical Information Mart for Intensive Care
ICU	intensive care unit
TG	triglycerides
FBG	fasting blood glucose
CHF	congestive heart failure
MI	myocardial infarction
PVD	peripheral vascular disease
SBP	systolic blood pressure
DBP	diastolic blood pressure
MBP	mean blood pressure
RR	respiratory rate
HR	heart rate
ACEI/ARB	angiotensin converting enzyme inhibitor/angiotensin receptor blocker
CCB	calcium channel blocker
BUN	blood urea nitrogen
PT	prothrombin time
PTT	partial prothrombin time
RBC	red blood cell
WBC	white blood cell
GCS	Glasgow coma scale
SOFA	sequential organ failure assessment
SAPSII	simplified acute physiology score
LOS	length of stay
WHO	World Health Organization
SD	standard deviation
ANOVA	analysis of variance
IQR	interquartile range
KM	Kaplan–Meier
RCS	restricted cubic spline
HRs	hazard ratios
CI	confidence intervals
MHO	metabolically healthy obesity
MUO	metabolically abnormal obesity
ROS	reactive oxygen species
FFA	free fatty acid
GSH	glutathione
NADPH	nicotinamide adenine dinucleotide phosphate
AT	adipose tissue
