# Supplementary material for: Triglyceride–Glucose Index Modifies Mortality Risk Across Body Mass Index Strata in Critically Ill Patients: A Retrospective Cohort Analysis of the MIMIC-IV Database
Source: J Clin Med. 2026 May 11;15(10):3685. doi: 10.3390/jcm15103685 (PMC13207182; doi:10.3390/jcm15103685)
Supplement: Supplementary file 1 [file jcm-15-03685-s001.zip › jcm-4274906-supplementary.pdf]

Supplemental Materials

Title: Triglyceride-glucose index modifies mortality risk across body mass index strata in critically ill patients: a retrospective cohort analysis of the MIMIC-IV database

Directory

Supplementary Figure S1: Kaplan-Meier survival analysis curves for 365-day all-cause mortality **2**

Supplementary Figure S2: Relationship between body mass index and 365-day all-cause mortality stratified by race **3**

Supplementary Figure S3 Relationship between body mass index and 365-day all-cause mortality stratified by sex **4**

Supplementary Table S1: Baseline characteristics **5**

Supplementary Table S2: COX regression analysis of 365-day all-cause mortality stratified by TyG dichotomy **8**

Supplementary Table S3: COX regression analysis of 365-day all-cause mortality in the combined obesity group **9**

Supplementary Table S4: COX regression analysis of 180-day all-cause mortality rate **10**

Supplementary Table S5: COX regression analysis of 365-day all-cause mortality in patients with cardiovascular diseases **11**

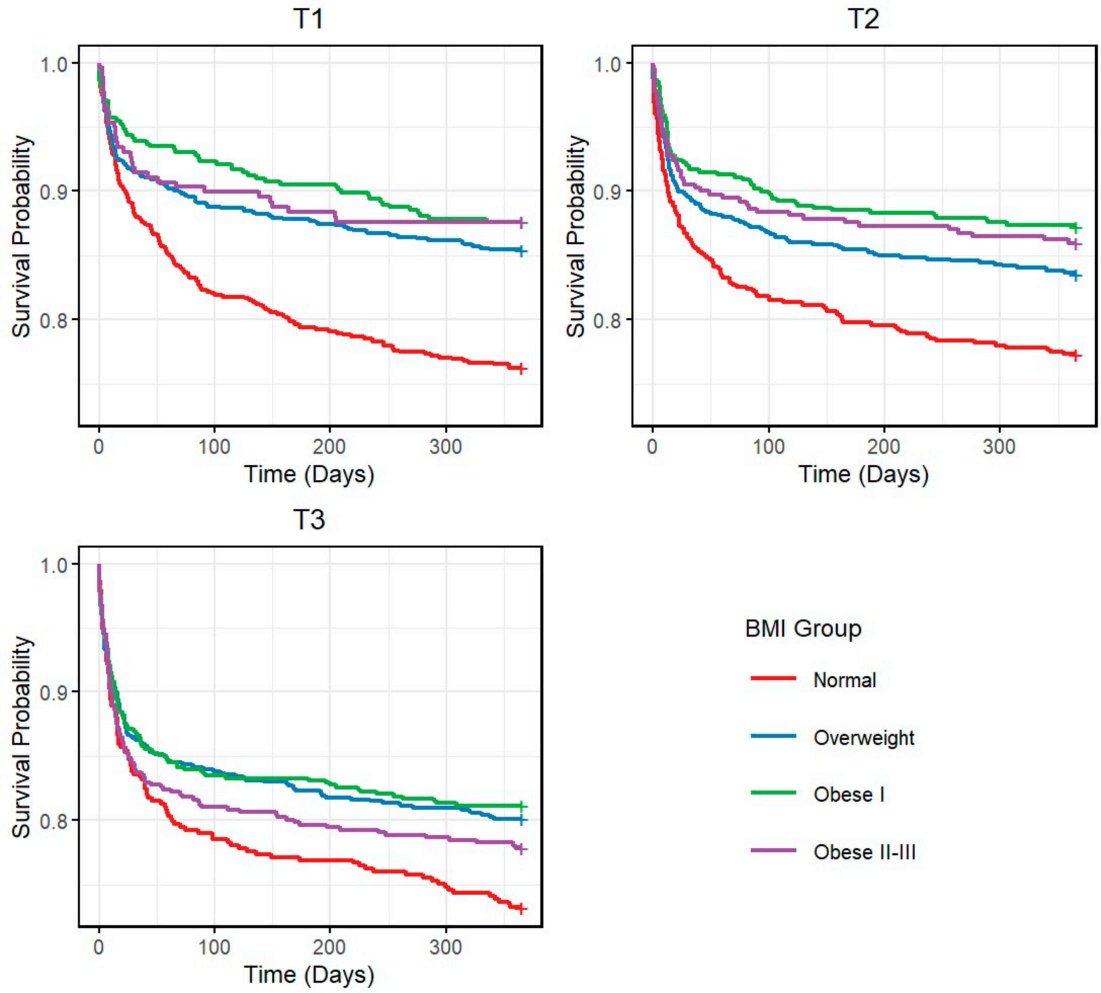

Supplementary Figure S1 Kaplan-Meier survival analysis curves for 365-day all-cause mortality

The participants were categorized into T1, T2, and T3 groups based on the tertiles of TyG

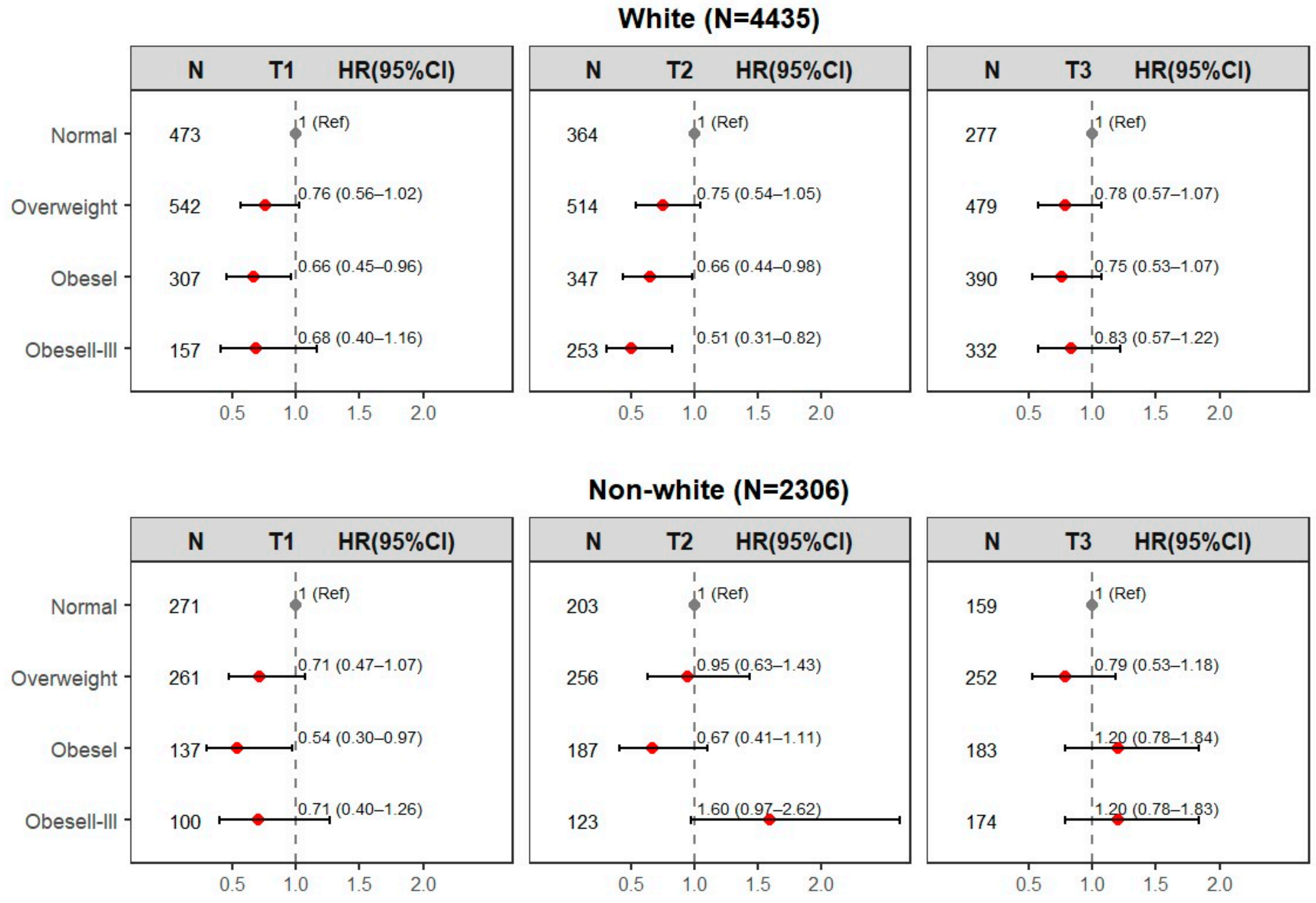

Supplementary Figure S2 Relationship between body mass index and 365-day all-cause mortality stratified by race.The black circle represents the reference group (Normal BMI; HR = 1.0), and the red circles represent the hazard ratios (HRs) for the comparison groups (Overweight, Obese).

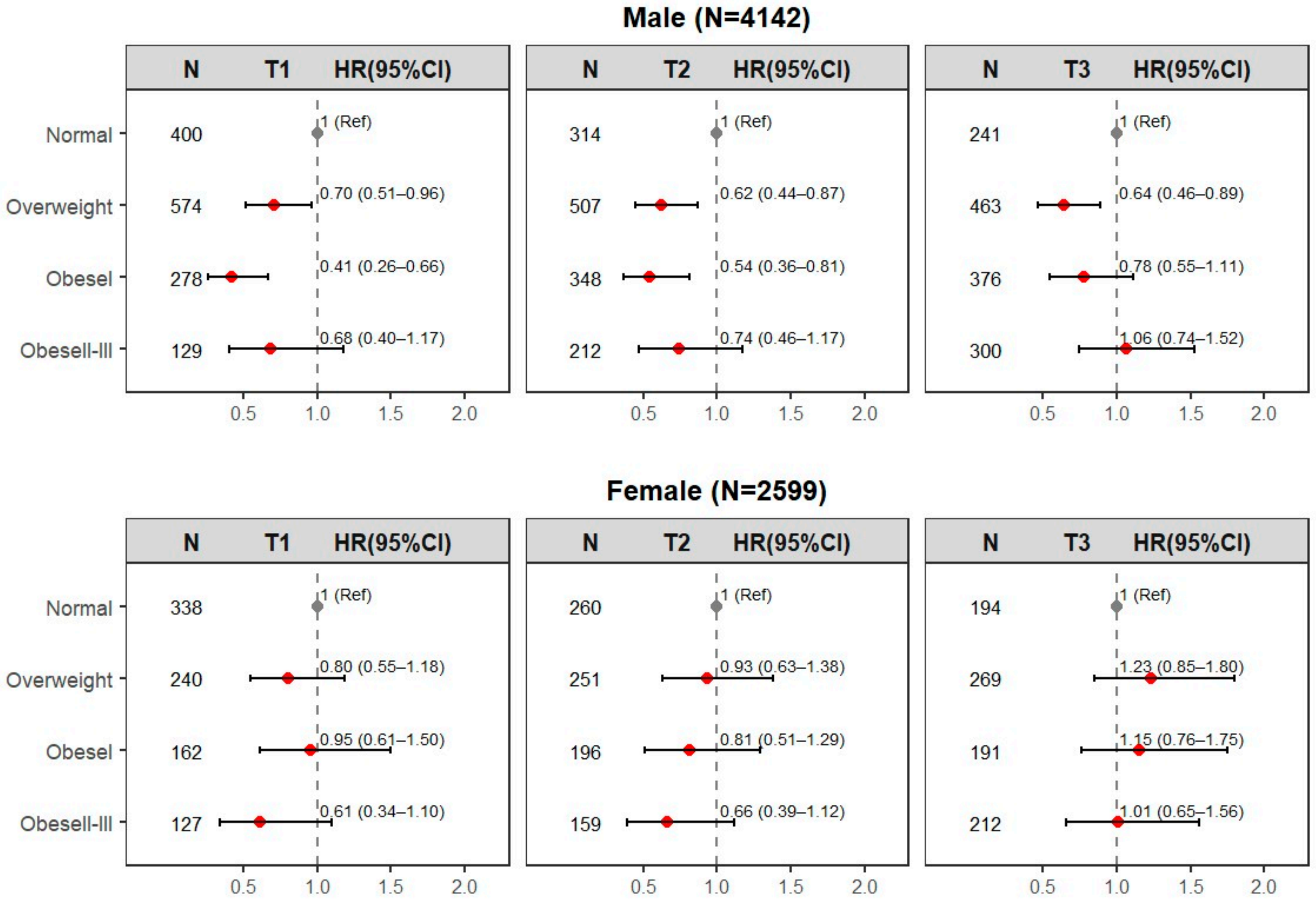

Supplementary Figure S3 Relationship between body mass index and 365-day all-cause mortality stratified by sex.The black circle represents the reference group (Normal BMI; HR = 1.0), and the red circles represent the hazard ratios (HRs) for the comparison groups (Overweight, Obese).

Supplementary Table S1 Baseline characteristics

**Variable**	**T1**<br>**＜8.62(*N* = 2311)**	**T2**<br>**(8.62-9.20) (*N* = 2311)**	**T3**<br>**＞9.20 (*N* = 2311)**	**P-value**
**Demographics**				
Age, years	69.00 (56.50, 79.00)	67.00 (56.00, 77.00)	62.00 (49.00, 72.00)	<0.001
Sex, male	1370 (59.3)	1388 (60.1)	1462 (63.3)	0.013
Race, white	1547 (66.9)	1590 (68.8)	1422 (61.5)	<0.001
Weight, Kg	77.20 (65.70, 90.00)	81.20 (67.50, 96.25)	86.10 (72.90, 102.00)	<0.001
Height, cm	170.00 (163.00, 178.00)	170.00 (163.00, 178.00)	170.00 (163.00, 178.00)	0.002
BMI, Kg/m2	26.73 (23.40, 30.80)	28.16 (24.37, 32.41)	29.44 (25.64, 34.28)	<0.001
**Burden of disease**, n (%)				
Cerebrovascular disease	475 (20.6)	503 (21.8)	406 (17.6)	0.001
Chronic pulmonary disease	525 (22.7)	493 (21.3)	514 (22.2)	0.515
CHF	702 (30.4)	648 (28.0)	551 (23.8)	<0.001
Hypertension	1086 (47.0)	1079 (46.7)	1044 (45.2)	0.414
Mild liver disease	190 (8.2)	157 (6.8)	225 (9.7)	0.001
MI	488 (21.1)	512 (22.2)	489 (21.2)	0.623
PVD	304 (13.2)	326 (14.1)	296 (12.8)	0.406
Peptic ulcer disease	69 (3.0)	50 (2.2)	64 (2.8)	0.195
Rheumatic disease	89 (3.9)	74 (3.2)	94 (4.1)	0.269
Renal disease	340 (14.7)	332 (14.4)	330 (14.3)	0.907
Stroke	269 (11.6)	252 (10.9)	169 (7.3)	<0.001
**Vital sign**				
SBP, mmHg	113.85 (105.73, 124.09)	113.96 (106.19, 124.78)	114.08 (106.19, 123.96)	0.515
DBP, mmHg	61.92 (55.55, 69.58)	62.29 (56.36, 69.87)	63.39 (57.53, 70.11)	<0.001
MBP, mmHg	76.97 (71.51, 84.82)	77.81 (72.19, 84.84)	78.19 (73.13, 85.13)	<0.001

RR, times/min	18.17 (16.31, 20.52)	18.47 (16.62, 21.14)	19.70 (17.25, 23.14)	<0.001
HR, beats/min	80.20 (72.23, 90.66)	81.71 (73.38, 92.02)	85.38 (75.53, 98.51)	<0.001
**Medication**, n (%)				
ACEI/ARB	798 (34.5)	814 (35.2)	699 (30.2)	0.001
Clopidogrel	273 (11.8)	303 (13.1)	304 (13.2)	0.298
Ticagrelor	60 (2.6)	67 (2.9)	72 (3.1)	0.569
Beta blockers	474 (20.5)	549 (23.8)	601 (26.0)	<0.001
CCB	508 (22.0)	594 (25.7)	552 (23.9)	0.012
Diuretics	1516 (65.6)	1616 (69.9)	1629 (70.5)	<0.001
Amiodarone	521 (22.5)	491 (21.2)	402 (17.4)	<0.001
Insulin	1376 (59.5)	1468 (63.5)	1638 (70.9)	<0.001
Statin	1383 (59.8)	1421 (61.5)	1285 (55.6)	<0.001
Heparin	1885 (81.6)	1958 (84.7)	2079 (90.0)	<0.001
Warfarin	561 (24.3)	513 (22.2)	408 (17.7)	<0.001
**Laboratory data**				
SpO_2, %	97.52 (96.12, 98.73)	97.42 (96.03, 98.59)	97.14 (95.68, 98.46)	<0.001
Anion gap, mmol/L	13.00 (11.00, 15.00)	13.00 (11.00, 16.00)	14.00 (12.00, 17.00)	<0.001
Calcium, mg/dl	8.40 (7.90, 8.90)	8.40 (7.90, 8.90)	8.30 (7.70, 8.80)	<0.001
Bicarbonate, mg/dl	23.00 (21.00, 26.00)	23.00 (21.00, 25.00)	22.00 (19.00, 25.00)	<0.001
BUN, mg/dl	17.00 (12.00, 24.00)	17.00 (12.00, 24.00)	18.00 (13.00, 27.00)	<0.001
Chloride, mEq/L	105.00 (101.00, 107.00)	105.00 (102.00, 108.00)	104.00 (101.00, 107.00)	0.002
Creatinine, mg/dl	0.90 (0.70, 1.20)	0.90 (0.70, 1.20)	1.00 (0.80, 1.40)	<0.001
Sodium, mEq/L	139.00 (136.00, 141.00)	139.00 (136.00, 141.00)	138.00 (136.00, 141.00)	0.102
Potassium, mEq/L	4.10 (3.80, 4.50)	4.20 (3.80, 4.50)	4.20 (3.80, 4.60)	0.025
Glucose, mg/dl	107.00 (93.00, 122.00)	121.00 (106.00, 141.00)	141.00 (117.00, 177.00)	<0.001
PT, s	14.20 (12.50, 16.70)	14.00 (12.50, 16.10)	13.80 (12.30, 15.90)	<0.001
PTT, s	31.30 (27.50, 39.00)	30.80 (27.30, 37.70)	30.30 (26.80, 37.45)	<0.001

TG, mg/dl	73.00 (58.00, 89.00)	120.00 (100.00, 142.00)	215.00 (165.00, 291.00)	<0.001
RBC, m/uL	3.60 (3.05, 4.18)	3.72 (3.16, 4.29)	3.80 (3.22, 4.40)	<0.001
Platelet, K/uL	180.00 (133.00, 239.00)	186.00 (142.00, 249.00)	199.00 (144.00, 260.50)	<0.001
WBC, K/uL	9.70 (7.10, 13.30)	11.00 (8.00, 15.10)	12.30 (8.60, 17.10)	<0.001
GCS	15.00 (14.00, 15.00)	15.00 (14.00, 15.00)	15.00 (14.00, 15.00)	<0.001
SOFA	4.00 (2.00, 6.00)	4.00 (2.00, 7.00)	5.00 (3.00, 8.00)	<0.001
SAPSII	33.00 (26.00, 42.00)	34.00 (26.00, 42.00)	36.00 (27.00, 46.00)	<0.001
TyG	8.31 (8.08, 8.48)	8.90 (8.76, 9.04)	9.59 (9.37, 9.97)	<0.001
**Length of stay (LOS)**				
LOS in hospital	7.88 (4.99, 13.48)	8.68 (5.18, 15.36)	10.73 (5.98, 20.98)	<0.001
LOS in ICU	2.32 (1.28, 5.07)	2.87 (1.40, 6.25)	4.35 (1.85, 10.95)	<0.001
**Outcome**				
180-day mortality, n (%)	346 (15.0)	354 (15.3)	438 (19.0)	<0.001
365-day mortality, n (%)	410 (17.7)	401 (17.4)	492 (21.3)	0.001

BMI, body mass index; CHF, congestive heart failure; MI, myocardial infarction; PVD, peripheral vascular disease; SBP, systolic blood pressure; DBP, diastolic blood pressure; MBP, mean blood pressure; RR, respiratory rate; HR, heart rate; ACEI/ARB, angiotensin converting enzyme inhibitor/angiotensin receptor blocker; CCB, calcium channel blockers; BUN, blood urea nitrogen; PT, prothrombin time; PTT, partial prothrombin time; TG, triglyceride; RBC, red blood cell; WBC, white blood cell; GCS, Glasgow coma scale; SOFA, sequential organ failure assessment; SAPSII, simplified acute physiology score; TyG, triglyceride-glucose

Supplementary Table S2 COX regression analysis of 365-day all-cause mortality stratified by TyG dichotomy

	BMI group	Event/N	HR（95%CI）	P-value
Q1(N=3371)	Normal	252/1035	Reference	-
	Overweight	171/1195	0.68 (0.55-0.82)	＜0. 001
	Obese I	84/704	0.60 (0.46-0.77)	＜0. 001
	Obese II-III	52/437	0.61(0.45-0.84)	0.002
Q2(N=3370)	Normal	170/712	Reference	-
	Overweight	218/1109	0.91(0.74-1.12)	0.375
	Obese I	147/847	0.93 (0.74-1.17)	0.536
	Obese II-III	145/702	1.09(0.86-1.38)	0.461

Adjusted for Age, Sex, Race, SOFA, SAPSII, PT, BUN, WBC, Potassium, Hypertension, Stroke, Renal disease, Insulin, Statin, and Diuretics

Supplementary Table S3 COX regression analysis of 365-day all-cause mortality in the combined obesity group

	BMI group	Event/N	HR（95%CI）	P-value
T1(N=2311)	Non-overweight/obese	206/823	Reference	-
	Overweight	117/798	0.71 (0.56-0.90)	0.003
	Obese	87/690	0.61 (0.47-0.79)	< 0.001
T2(N=2311)	Non-overweight/obese	157/643	Reference	-
	Overweight	125/764	0.71 (0.56-0.91)	0.007
	Obese	119/904	0.64(0.50-0.83)	< 0.001
T3(N=2311)	Non-overweight/obese	123/473	Reference	-
	Overweight	147/742	0.85(0.67-1.09)	0.196
	Obese	222/1096	1.01(0.80-1.28)	0.928

Adjusted for Age, Sex, Race, SOFA, SAPSII, PT, BUN, WBC, Potassium, Hypertension, Stroke, Renal disease, Insulin, Statin, and Diuretics

Supplementary Table S4 COX regression analysis of 180-day all-cause mortality rate

	BMI group	Event/N	HR（95%CI）	P-value
T1(N=2247)	Normal	153/548	Reference	-
	Overweight	98/804	0.72 (0.55-0.94)	0.014
	Obese I	42/444	0.56(0.39-0.80)	0.007
	Obese II-III	30/258	0.74(0.49-1.10)	0.138
T2(N=2247)	Normal	116/573	Reference	-
	Overweight	111/765	0.78 (0.59-1.01)	0.064
	Obese I	62/539	0.67 (0.49-0.92)	0.015
	Obese II-III	47/370	0.77(0.54-1.10)	0.152
T3(N=2247)	Normal	100/433	Reference	-
	Overweight	130/735	0.85(0.65-1.10)	0.221
	Obese I	95/568	0.95(0.71-1.27)	0.714
	Obese II-III	104/511	1.11(0.83-1.50)	0.475

Adjusted for Age, Sex, Race, SOFA, SAPSII, PT, BUN, WBC, Potassium, Hypertension, Stroke, Renal disease, Insulin, Statin, and Diuretics

Supplementary Table S5 COX regression analysis of 365-day all-cause mortality in patients with cardiovascular diseases

	BMI group	Event/N	HR（95%CI）	P-value
T1(N=1702)	Normal	144/548	Reference	-
	Overweight	103/615	0.82 (0.63-1.07)	0.139
	Obese I	45/352	0.63(0.44-0.89)	0.009
	Obese II-III	25/187	0.68(0.44-1.06)	0.088
T2(N=1702)	Normal	109/410	Reference	-
	Overweight	107/591	0.81 (0.61-1.06)	0.127
	Obese I	53/428	0.60 (0.43-0.85)	0.004
	Obese II-III	39/273	0.74(0.51-1.10)	0.130
T3(N=1702)	Normal	89/312	Reference	-
	Overweight	111/547	0.86(0.64-1.14)	0.288
	Obese I	80/448	0.90(0.66-1.23)	0.514
	Obese II-III	89/395	1.09(0.79-1.49)	0.616

Patients with cardiovascular disease including myocardial infarction, congestive heart failure, cerebrovascular disease, stroke and hypertension

Adjusted for Age, Sex, Race, SOFA, SAPSII, PT, BUN, WBC, Potassium, Hypertension, Stroke, Renal disease, Insulin, Statin, and Diuretics
